# Maternal Diet, Metabolic State, and Inflammatory Response Exert Unique and Long-Lasting Influences on Offspring Behavior in Non-Human Primates

**DOI:** 10.3389/fendo.2018.00161

**Published:** 2018-04-23

**Authors:** Jacqueline R. Thompson, Hanna C. Gustafsson, Madison DeCapo, Diana L. Takahashi, Jennifer L. Bagley, Tyler A. Dean, Paul Kievit, Damien A. Fair, Elinor L. Sullivan

**Affiliations:** ^1^Division of Neuroscience, Oregon National Primate Research Center, Beaverton, OR, United States; ^2^Division of Cardiometabolic Health, Oregon National Primate Research Center, Beaverton, OR, United States; ^3^Department of Psychiatry, Oregon Health and Science University, Portland, OR, United States; ^4^Department of Behavioral Neuroscience, Oregon Health and Science University, Portland, OR, United States; ^5^Department of Human Physiology, University of Oregon, Eugene, OR, United States

**Keywords:** behavior, development, macrophage-derived chemokine (MDC), maternal, adiposity, Western-style, diet, inflammation, macaque

## Abstract

Nutritional status influences brain health and gestational exposure to metabolic disorders (e.g. obesity and diabetes) increases the risk of neuropsychiatric disorders. The aim of the present study was to further investigate the role of maternal Western-style diet (WSD), metabolic state, and inflammatory factors in the programming of Japanese macaque offspring behavior. Utilizing structural equation modeling, we investigated the relationships between maternal diet, prepregnancy adiposity, third trimester insulin response, and plasma cytokine levels on 11-month-old offspring behavior. Maternal WSD was associated with greater reactive and ritualized anxiety in offspring. Maternal adiposity and third trimester macrophage-derived chemokine (MDC) exerted opposing effects on offspring high-energy outbursts. Elevated levels of this behavior were associated with low maternal MDC and increased prepregnancy adiposity. This is the first study to show that maternal MDC levels influence offspring behavior. We found no evidence suggesting maternal peripheral inflammatory response mediated the effect of maternal diet and metabolic state on aberrant offspring behavior. Additionally, the extent of maternal metabolic impairment differentially influenced chemokine response. Elevated prepregnancy adiposity suppressed third trimester chemokines, while obesity-induced insulin resistance augmented peripheral chemokine levels. WSD also directly increased maternal interleukin-12. This is the first non-human primate study to delineate the effects of maternal diet and metabolic state on gestational inflammatory environment and subsequent offspring behavior. Our findings give insight to the complex mechanisms by which diet, metabolic state, and inflammation during pregnancy exert unique influences on offspring behavioral regulation.

## Introduction

As trends toward global urbanization rise, a Western-style diet (WSD) is becoming increasingly common worldwide. The WSD is calorically dense and highly palatable, characterized by a high proportion of sugar and fat (specifically saturated fats). Increased consumption of a WSD is altering public health concerns, as the risk of nutrient deficiency and infectious diseases is falling while the prevalence of cardiometabolic diseases grows (1). Primary factors identified as contributing to global deaths are characteristics of a WSD (diet high in sodium, low in whole grain, and high in processed meat) or consequences of WSD consumption (hypertension, elevated fasting plasma glucose, high total cholesterol, and increased body mass index) (2). Current figures indicate that the global prevalence of obesity has increased dramatically in the past decade and at most recent estimates is 13.7% in adult men and 21.5% in adult women (3, 4).

It is well established that maternal diet and metabolic state during pregnancy alter future risk of metabolic disease in offspring (5). Gestational factors such as increased maternal weight gain, obesity, and impaired glucose metabolism can likewise impact offspring neurodevelopment, increasing the risk of neuropsychiatric disorders (6–8). Similarly, perinatal nutrition is an important determinant of children’s neural outcome, and exposure to aspects of a WSD during the perinatal period increases risk of neuropsychiatric disorders such as autism spectrum disorder (ASD), attention-deficit hyperactivity disorder (ADHD), and schizophrenia (9–11).

While human studies highlight the importance of the early environment on long-term offspring outcomes, animal models are integral to further define the mechanisms that underlie aberrant development. Preclinical animal models have demonstrated fetal exposure to diet-induced obesity results in neural reprogramming in multiple brain regions and neuroendocrine systems (12–15). Importantly, these diet-induced neurodevelopmental and behavioral aberrations are associated with altered neuroinflammatory response (16–18). Inflammation is believed to be crucial to perinatal behavioral programming (19, 20), and human studies support a link between increased inflammatory factors and neurodevelopmental disorders (21–23).

Inflammation is a particularly attractive mechanism for fetal programming due to its extensive involvement in obesity, diabetes, and hypertension (24–27). Inflammation is a chronic or severe imbalance between initiative and reparative immune responses, often generalized by the two predominant macrophage phenotypes, M1 (classically activated and proinflammatory) and M2 (alternatively activated and anti-inflammatory). Chronic metabolic conditions induce systemic inflammation and alter the microenvironment, resulting in hypertrophic growth, oxidative stress, hypoxia, and altered lipid regulation and glucose metabolism (26). These factors drive proinflammatory response in adipose tissue, altering the resident macrophage population (28). In addition to M1 and M2 forms, a metabolically activated adipose tissue macrophage (ATM) phenotype was found which is capable of switching between pro- or anti-inflammatory functions depending on factors such as the extent of obesity progression and adipocyte death (29, 30).

The inflammatory milieu has a unique function during pregnancy, with the interplay between pro- and anti-inflammatory responses helping to drive successful pregnancy outcomes (31–33). The presence of chemokine proteins can be particularly important as the chemotaxis gradient generated helps peripheral cells direct immune response to the maternal–fetal boundary (34). The inflammatory burden resulting from a single immunocompromised state, such as pregnancy, can be distinctively altered when compounded with chronic inflammation. Elevated adiposity and abnormal glucose metabolism prior to—or during—pregnancy can limit the ability to accommodate fetal demands and are associated with unique and targeted changes in inflammation during gestation (35–37). The inflammation present *in utero* can be transferred from mother to fetus (38). Even in the absence of inflammatory changes in maternal plasma, exposure to WSD-induced obesity and insulin resistance increased fetus-derived proinflammatory response (39). The inflammatory response is believed to be an integral mechanistic component that links diet, metabolic state, and neurodevelopment, with altered inflammatory pathways during pregnancy potentially contributing to fetal programming.

While some studies have begun to examine the distinct programming effects of maternal diet from metabolic state, this differentiation remains largely unaddressed, particularly with respect to offspring behavior. The goal of the current study is to use maternal diet, metabolic state, and inflammation during pregnancy to model how select perinatal environmental factors independently contribute to offspring behavioral response in Japanese macaques. We hypothesize that WSD-induced programming of offspring behavior is mediated by inflammation and that metabolically activated immune activity is intimately involved in this response. Due to the longstanding nature of the project, this study is uniquely suited to investigate whether maternal factors have independent effects on offspring development. This is the first non-human primate study to examine the discrete effects of chronic WSD consumption, maternal metabolic state, and gestational inflammatory profile on offspring behavior.

## Materials and Methods

### Animal Demographics

All animal procedures were in accordance with National Institutes of Health guidelines on the ethical use of animals and were approved by the Oregon National Primate Research Center (ONPRC) Institutional Animal Care and Use Committee.

Adult Japanese macaques (*Macaca fuscata)* were housed in indoor/outdoor pens containing 4–12 individuals (male/female group ratio of 1-2/3-10). Males were either intact or vasectomized depending on the reproduction requirements for the group. Animals were given *ad libitum* access to water and each breeding group was assigned to an experimental diet, either the control (CTR) or WSD. Both males and females within a group exclusively consumed the designated diet as the majority of their calorie intake, supplemented by daily enrichment of fruits or vegetables. The WSD (TAD Primate Diet no. 5LOP, Test Diet, Purina Mills) provides approximately 36.6% of calories from fat, which is in line with the fat and saturated fat content of the typical Western-style, American diet. Alternatively, the CTR diet (Monkey Diet no. 5000; Purina Mills) provides approximately 14.7% of calories from fat. The carbohydrate sources differed between the two diets, with sugars (primarily sucrose and fructose) comprising 18.94% of the WSD but only 3.14% of the CTR diet. All animals fed the WSD were also given calorically dense treats once per day (40).

Adult females had consumed the WSD for at least 1 year before producing offspring considered in this study. Females were allowed to breed with intact males and were sedated two to three times during pregnancy for fetal dating and third trimester measures (described below). Pregnant females gave birth naturally in their social groups and most offspring began independently ingesting the maternal diet by 4 months of age; by 6 months of age this diet was their primary food source. At a mean age of 7.97 ± 0.08 months, the offspring were weaned and relocated to group-housing with 6–10 similarly aged juveniles and 1–2 unrelated adult females. While half of the offspring were kept on their mother’s diet, the other half switched diets at weaning. Sample size varied between measures as the data was collected over nine years and some procedures were added in later years. The sample sizes for each group and the average age of the animals for the various measures are described in Table 1.

**Table 1:** Animal numbers and ages for procedures.

Maternal measures	Age (years), mean ± SE	Days pre-/postconception, mean ± SE	*N* (per diet group)
Prepregnancy adiposity	8.96 ± 0.24	87.89 ± 11.35	116 (CTR = 61, WSD = 55)
Third trimester IAUC	9.47 ± 0.22	127.62 ± 0.66	114 (CTR = 46, WSD = 68)
Third trimester inflammatory markers	9.27 ± 0.24	123.36 ± 1.43	117 (CTR = 52, WSD = 65)

**Juvenile measures**	**Age (months), mean ± SE**	***N* (per diet group)**	

Behavioral assessment	10.86 ± 0.02	74 (Maternal CTR = 32, WSD = 42; Postweaning CTR = 44, WSD = 30; M = 37, F = 37)	

*Due to the long-term nature of the project, sample sizes varied between procedures. Non-pregnant measures include days preconception, and third trimester measures include days postconception. Mean ± SE were as follows: prepregnancy adiposity (CTR = 19.96% ± 1.15% body fat; WSD = 26.09 ± 1.41% body fat) and third trimester IAUC (CTR = 7649.88 ± 1465.53 μIU/mL min; WSD = 11095.83 ± 1114.99 μIU/mL min). For juvenile behavior, diet groups indicate maternal diet and postweaning diet*.

*SE, standard error of the estimate; IAUC, insulin area under the curve; CTR, control diet; WSD, Western-style diet; M, male offspring; F, female offspring*.

### Fetal Dating

Between October and April, adult females were regularly assessed for pregnancy. Females were removed from the group and sedated with either Ketathesia (5–15 mg/kg IM; ketamine HCl, Henry Schein Animal Health, Dublin, OH, USA) or Telazol (3 mg/kg IM; tiletamine HCl and zolazepam HCl, Zoetis Inc., Kalamazoo, MI, USA), positioned in dorsal recumbency, and checked for pregnancies *via* ultrasound. The uterus was scanned from cranial to caudal and left to right. If an embryo was identifiable, biparietal diameter was measured at the widest part of the cranium, and if ≥10.9 mm, this measure was used to estimate fetal age. If biparietal diameter was less than 10.9 mm, then fetal greatest length was measured from the top of the head to the base of the tail, as viewed in the sagittal plane. In practice, these measurements have been shown to accurately predict gestational age between gestational day (GD) 21 and GD 172 in Japanese macaques. Occasionally pregnancy could be observed but embryonic measurements could not be made due to early gestational age. In these cases, the animal would undergo another pregnancy check approximately one month later.

Fetal gestational age at ultrasound was used to estimate date of conception. Prepregnancy adiposity was evaluated with respect to days prior to conception, and procedures conducted during pregnancy were performed at the third trimester, scheduled to be 120 days after estimated conception (approximately 50 days before parturition) (Table 1). Our third trimester procedures occurred an average of 47.51 ± 0.75 days before parturition, further validating the accuracy of the fetal dating parameters.

### Maternal Prepregnancy Adiposity

Prior to each pregnancy, dual-energy X-ray absorptiometry scans were completed to determine maternal body composition. Animals were sedated with Telazol (3–8 mg/kg IM) before being positioned prone on the bed of a Hologic QDR Discovery scanner (Hologic, Bedford, MA, USA). The “Adult Whole Body” scan mode was used and Hologic QDR Software version 12.6.1 was used to calculate percent body fat. The mean ± standard error of prepregnancy adiposity for each diet group is as follows: CTR = 19.96 ± 1.15% body fat; WSD = 26.09 ± 1.41% body fat.

### Maternal Insulin Response

Intravenous glucose tolerance tests were performed on pregnant adult females during the third trimester. Animals were fasted overnight and the following morning. Initial sedation was accomplished using Telazol (3–8 mg/kg IM), and if needed additional anesthesia was accomplished with Ketathesia (3–10 mg/kg IM). A catheter was placed in both left and right saphenous veins. A 3–8 mL baseline blood sample was collected just prior to the glucose infusion, half of which was placed in a sodium heparin tube and stored on ice. A bolus of 50% Dextrose solution (Durvet, Inc., Blue Springs, MI, USA) was administered at a dose of 0.6 g/kg through one of the saphenous catheters. In order to determine changing insulin levels after infusion, 0.5 mL whole blood samples were drawn though the opposite catheter at predetermined intervals and placed in heparinized tubes on ice. After each sample, 0.5 mL of heparinized saline was flushed through the catheter to prevent clotting. Blood samples were obtained at baseline and 1, 3, 5, 10, 20, 40, and 60 min after infusion.

Postprocedure, all heparinized blood samples were centrifuged at 2400 RPM for 20 min at 4°C. Plasma supernatant was aliquoted and stored at −80°C until time of assay. Plasma concentrations of insulin were measured by the Endocrine Technologies Support Core (ETSC) at the ONPRC using a chemiluminescence-based automatic clinical platform (Roche Diagnostics Cobas e411, Indianapolis, IN, USA) (41). The range of the insulin assay was 0.2–1,000 μIU/mL. The intra- and inter-assay variations were less than 7%. All quality controls and calibrations provided by the company, as well as ETSC monkey serum controls, were analyzed before each use for hormonal measurements in samples. From these insulin measures, area under the curve was calculated from zero using GraphPad Prism Version 6 software (Graphpad Software, Inc., La Jolla, CA, USA). Insulin area under the curve (IAUC) after exogenous glucose infusion represented an individual’s insulin response, with exaggerated IAUC indicating insulin resistance. The mean ± standard error of third trimester IAUC for each diet group is as follows: CTR = 7649.88 ± 1465.53 μIU/mL min; WSD = 11095.83 ± 1114.99 μIU/mL min.

### Cytokine Samples and Processing

Plasma samples were collected from adult females at the third trimester (described above). Baseline plasma supernatant was aliquoted and stored at −80°C until time of assay. Plasma cytokine levels were determined using a monkey 29-plex cytokine panel (ThermoFisher Scientific, Waltham, MA, USA) following the manufacturer’s instructions. Two plates were used to complete this analysis, both originating from the same lot (#1833398A). Concentrations of each cytokine were calculated from a standard control curve. Samples were analyzed on a Milliplex Analyzer (EMD Millipore, Billerica, MA, USA) bead sorter with XPonent Software version 3.1 (Luminex, Austin, TX, USA). Data were calculated using Milliplex Analyst software version 5.1 (EMD Millipore). The inter-assay CV and lower limit of quantification (LLOQ) for each assay are listed in Table 2. Only inflammatory markers for which greater than 80% of the samples reported values above LLOQ were analyzed in this study. Values that measured below the LLOQ were replaced with the square root of two-thirds the LLOQ for analysis. EGF was not included in subsequent analysis because it was the only quantifiable growth factor and not involved in the cell signaling pathways represented by other inflammatory markers. Two adult females were excluded from this measure because their health records indicated they were clinically compromised shortly before or after blood sample collection. Two additional adult females were excluded because their samples were taken too close to the offspring’s birth (4 days before and 10 days after).

**Table 2:** Third trimester inflammatory markers.

Protein	Alternate name	LLOQ (pg/mL)	Percent of values above LLOQ	Interassay CV	Maternal WSD regression		Inflammatory burden latent variable		Chemokine latent variable	
					β	*p*-Value	Standardized factor loading	*p*-Value	Standardized factor loading	*p*-Value
**MCP-1**	CCL2	5.38	**100.00%**	2.72%	0.021	0.840	0.77	0.000	0.84	0.000
**RANTES**	CCL5	13.81	**100.00%**	3.60%	−0.050	0.544	0.55	0.000	0.51	0.003
**Eotaxin**	CCL11	0.59	**100.00%**	12.77%	−0.105	0.342	0.33	0.013	0.57	0.000
**I-TAC**	CXCL11	11.13	**100.00%**	6.35%	0.101	0.388	0.32	0.034	0.62	0.000
**MIP-1β**	CCL4	3.46	**99.14%**	2.42%	−0.046	0.686	0.44	0.001	0.34	0.000
**MDC**	CCL22	50.53	**97.41%**	3.70%	−0.236	0.012	0.48	0.000	0.43	0.000
**IP-10**	CXCL10	2.70	**87.93%**	2.81%	0.260	0.282	0.53	0.000	0.53	0.000
**IFN-γ**		1.46	**100.00%**	4.35%	−0.023	0.837	0.54	0.000		
**IL-1RA**		12.29	**100.00%**	3.28%	0.147	0.107	0.79	0.000		
**IL-12**		10.49	**100.00%**	2.71%	0.263	0.002	0.59	0.000		
**IL-1β**		2.58	**95.69%**	1.41%	−0.012	0.901	0.68	0.000		
**MIF**	GIF	15.47	**92.24%**	4.21%	−0.116	0.245	0.43	0.000		
**TNF-α**		3.98	**91.38%**	2.66%	0.082	0.454	0.48	0.000		
**IL-6**		1.23	**88.79%**	2.61%	0.042	0.717	0.33	0.002		
EGF		0.50	93.10%	4.57%						
VEGF	VPF	0.25	75.00%	3.67%						
HGF	SF	14.23	72.41%	4.52%						
IL-15		10.26	72.41%	4.53%						
G-CSF	CSF-3	48.44	67.24%	4.65%						
MIP-1α	CCL3	8.07	62.93%	3.29%						
IL-17		7.90	56.03%	4.56%						
IL-8	CXCL8	2.80	55.17%	2.96%						
IL-2		37.93	54.31%	3.67%						
MIG	CXCL9	21.42	37.07%	2.89%						
FGF-Basic	FGF-2	1.59	34.48%	3.62%						
IL-5		1.49	30.17%	6.73%						
GM-CSF	CSF-2	2.72	25.00%	2.89%						
IL-4		10.32	18.97%	5.61%						
CSIF	IL-10	4.06	12.93%	3.56%						

*The indicated proteins were measured using a monkey 29-plex cytokine panel. Bolded inflammatory markers reported >80% of the animals were above the LLOQ and were included in analysis. EGF was not further considered as it was the only growth factor represented after exclusionary LLOQ criteria. The results from the regression models where maternal diet was used to predict each individual marker are listed with β and *p* values; only MDC and IL-12 were significant (*p* < 0.05). Mean ± SE were as follows: MDC (CTR = 481.99 ± 66.11 pg/mL, WSD = 287.06 ± 28.17 pg/mL) and IL-12 (CTR = 252.46 ± 22.32 pg/mL, WSD = 512.74 ± 117.30 pg/mL). Parameters from the confirmatory factor analyses used to inform the construction of the inflammatory burden and chemokines latent variables are presented*.

*LLOQ, lower limit of quantification; CV, coefficient of variability; SE, standard error of the estimate; CTR, control diet; WSD, Western-style diet*.

### Juvenile Behavior Assessment

The 11-month-old juveniles were transferred from their groups to a cage in an adjacent room between 0800 and 0830, where they were housed until transport to behavioral testing suite. Animals were moved in a covered transfer box to the testing suite devoid of distractions and were immediately placed in a standard satellite primate cage (0.61 m × 0.69 m × 0.82 m) equipped with an attached tray (0.60 m × 0.23 m) level with the feed slot. The assessment time began as soon as the juveniles were placed in the testing cage and the transporter had left the room. All tests occurred between 0830 and 1200 and were videotaped through a one-way mirror.

The temperament assessment protocol has been previously described in detail (40). Briefly, the assessment began with a 10-min acclimation period followed by 2-min control period, where the monkey was alone in the behavior suite. A Human Intruder Test (HIT) was then performed, in which an unfamiliar female entered for three separate epochs, presenting potentially threatening social stimuli. The 2-min HIT epochs consisted of facial profile, prolonged eye contact, and prolonged eye contact with apple offer segments, each separated by 2-min control periods. Following the HIT, the juvenile underwent a novel object test consisting of two segments in which a novel object was placed on the tray attached to the testing cage. After the objects were placed, the animal was left to interact freely with the object for a determined length of time. The first object was a novel toy with eyes, which was presented to the animal for 5 min, after which time a novel food (pretzel) was placed on the tray and presented to the animal for 2 min. After the novel object test, the assessment ended and the animal was returned to their social group. Observers blind to offspring diet groups scored the behavior tests using continuous sampling methods with The Observer CT, Version 11 (Noldus Information Technology, Leesburg, VA, USA). Coded behaviors were categorized into behaviorally related and statistically correlated groups (Table 3), combined using the summed *z* score.

**Table 3:** Eleven-month temperament assessment behaviors.

**Inactive behaviors (%)**	

Withdrawn	Percent of time inactive in the back of the testing cage, relative to total test time

Crouch	Lowering entire ventral surface to the floor of the cage for ≥3 s, typically in response to a stimulus

Freeze	Suddenly stop all movement (often mid-stride) for ≥3 s. Typically in response to a threatening stimuli, such as the stranger. Ends with any change in body position, including minor head movement

Sleep	Stationary with eyes fully closed for ≥3 s, seemingly asleep

**Engaged behaviors**	

Jump (#)	To leap or jump as part of locomotion, with no limbs touching the cage

Explore (#)	Purposefully interacting with cage using mouth or hands

Eye contact (%)	Making direct eye contact for at least two consecutive seconds with stranger

Interact with objects (%)	Intentional physical contact with an object, using hands, feet, or mouth, including eating

**Reactive anxiety**	

Self-groom (#)	Pick through or lick own fur/skin or biting own nails. May include placing debris from coat into mouth

Shake (#)	Rapidly shake full body as if to remove water particles (resembles a wet dog shake)

Scratch (#)	Use fingers or toes to scratch own body

Fear grimace (#)	Draw back lips to display clenched or slightly parted teeth

Anxious vocalizations (#)	Coo, shriek, and chirp

Threat (#)	Threats directed at stranger, such as head thrusting or lunging

Open mouth threat (#)	Maintain eye contact with mouth open, with or without visible teeth

Lipsmack (%)	Rapidly open and close or purse lips while stranger in room

Teeth grind (%)	Clenching and grinding of teeth to produce an audible clicking/grinding sound

**Ritualized anxiety (%)**	

Stereotypy	Abnormal pattern of movement repeated ≥3 consecutive times

Abnormal movement	Abnormal repetitive movement that cannot be considered stereotypy either due to brief breaks in the pattern or only two repetitions

**High-energy outbursts (#)**	

Cage bite	Forcefully and aggressively bite the cage in a non-exploratory manner

Cage shake	Grasp and shake the bars of the cage

Escape	Forcefully attempting to push body through the cage, typically the feed slot or cage corner

Other active	Instances of atypical movement, commonly ritualized with continued recurrence throughout multiple periods of test. Often presented as single iterations of common stereotypic behavior (rock, jump/bounce, spin) or directed, forceful contact with side of cage, without being self-injurious

Roll	Rapid inversion of body into atypical position with persistent movement, often resulting in a roll onto cage floor

*The z scores of listed behaviors were summed into indicated categories based on functional and correlational relationships. A # indicates that the number of occurrences of behavior used as measure for designated category or behavior. A% indicates percent of total test time that the behavior occurred was used as measure for designated category or behavior*.

### Data Analysis

Data were analyzed using M*plus* 7.4 (42) using the robust maximum likelihood estimator. This estimator accommodates non-normal data by adjusting standard errors using the Huber-White sandwich estimator. Missing data were handled using full information maximum likelihood (43). Non-independence of observations (multiple offspring born to the same dam) was handled using M*plus*’ *cluster* command. Model fit was examined using the comparative fit index (CFI), the Tucker-Lewis index (TLI), and the root mean squared error of approximation (RMSEA). CFI and TLI values above 0.90 and RMSEA values below 0.06 indicate adequate model fit (44–46).

Cytokine levels were log transformed prior to data analysis. In an effort to comprehensively assess the role of inflammation in our model of fetal programming, we used three different approaches to address unique aspects of maternal inflammatory response during pregnancy: overall burden, response profiles, and individual, representative markers. The first approach involved creating an inflammatory burden latent variable that captured total maternal systemic inflammation. Informed by a confirmatory factor analysis, the inflammatory burden latent variable was indicated by each of the cytokines included in the current study: IFN-γ, TNF-α, MIF, interleukin (IL)-12, IL-1β, IL-1RA, IL-6, MCP-1 (CCL2), MIP-1β (CCL4), RANTES (CCL5), Eotaxin (CCL11), macrophage-derived chemokine (MDC) (CCL22), IP-10 (CXCL10), and I-TAC (CXCL11). Of those, all of the cytokines present were consistent with an M1 profile and the chemokines indicated a mixed phenotype. Because of this, the second approach created individual cytokine and chemokine latent variables, again using confirmatory factor analysis. The chemokine latent variable was indicated by MCP-1, MIP-1β, RANTES, Eotaxin, MDC, IP-10, and I-TAC. The cytokine latent variable was indicated by IFN-γ, TNF-α, MIF, IL-12, IL-1β, IL-1RA, and IL-6. Results from these factor analyses are described in the results section. The last approach we used was to examine individual inflammatory markers, consistent with most research in this area. We *a priori* decided to examine a limited number of individual protein markers in order to minimize the number of comparisons made. Because we had theoretical equipoise in terms of the selection of individual proteins (i.e., there is reason to believe that they all might be related to offspring behavioral development), we elected to use a data-driven approach to their selection. To identify inflammatory markers of interest to our investigation of offspring behavior and gestational environment, we ran (a) bivariate correlations among the protein markers and the offspring behavioral categories and (b) a series of main effect regression models in which maternal diet was used to predict each of the protein markers. From these results, we selected the most relevant inflammatory markers for consideration in addition to the latent variables.

**Figure 1:**
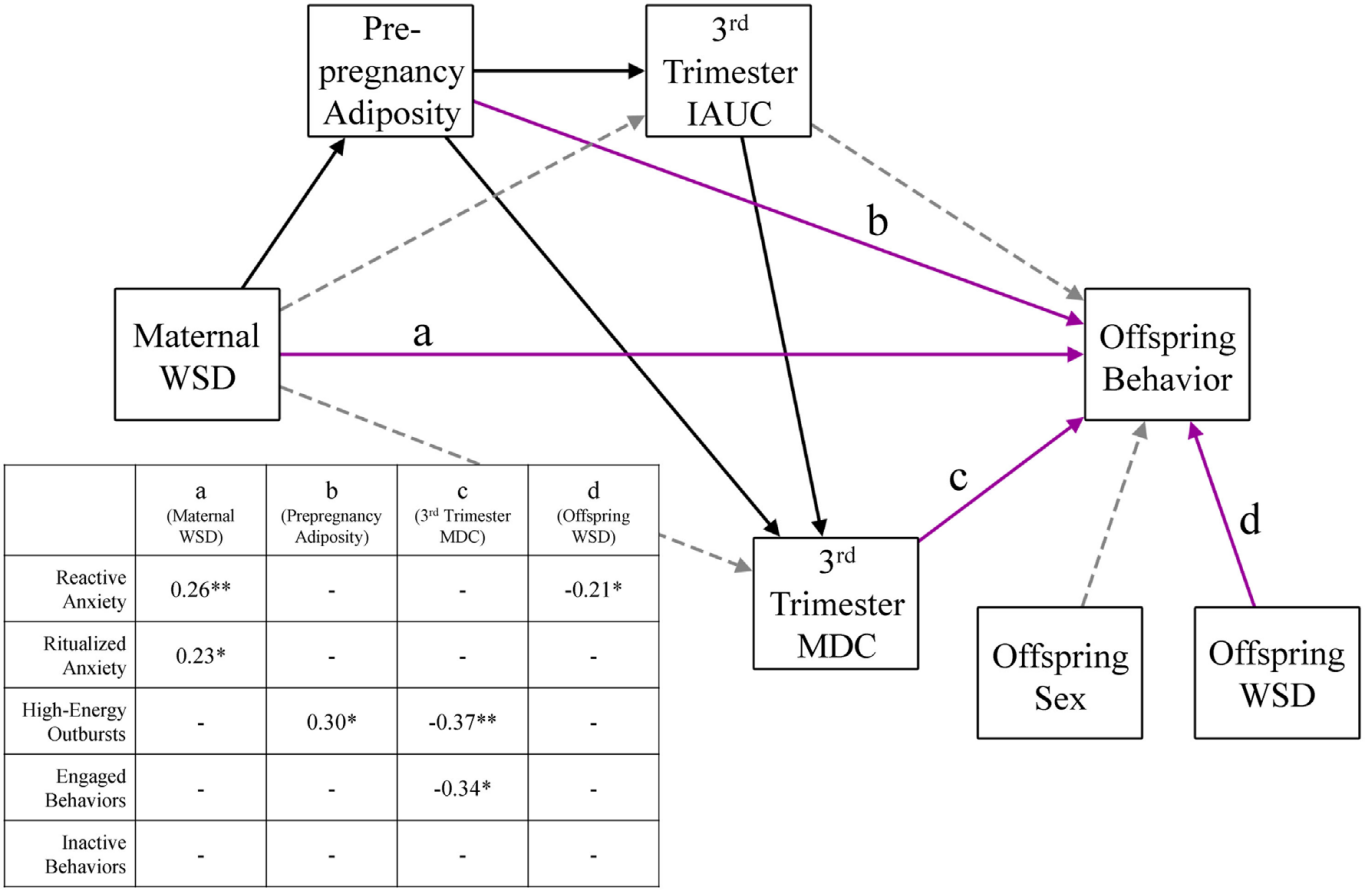
Perinatal environmental influences on offspring behavioral response. This figure presents the consolidated results of five different offspring behavior models, identical in every aspect except for the outcome behavior variable. Solid black lines were significant (*p* < 0.05) in every behavior model and gray dashed lines were not significant in any model. Magenta lines (labeled a–d) were significant in some behavior models; the inset table details the significant direct effects of the corresponding model. Paths were also estimated from maternal age to prepregnancy adiposity, third trimester IAUC, and third trimester MDC and were consistent across all behavior models but were not depicted for ease of readability. WSD, Western-style diet; IAUC, insulin area under the curve. *p < 0.05 and **p < 0.01.

Once we determined the inflammatory variables to include in analysis, a series of structural equation models (SEMs) were used to investigate the relative effects of maternal WSD, metabolic variables, and inflammation on each measure of offspring behavior. To limit the number of SEMs used, we first ran a series of main effect regression models to identify which of our maternal inflammation measures were associated with the five offspring behaviors. Only the measures of inflammation that were significantly (*p* < 0.05) associated with at least one behavior category after controlling for maternal age at birth were considered in further SEM analysis. Based on the results of these main effects models, SEMs were created for each measure of offspring behavior (i.e., engaged behaviors, inactive behaviors, reactive anxiety, ritualized anxiety, and high-energy outbursts). See Figure 1 for a visual depiction of these models. In each of these models, maternal inflammation was regressed on maternal WSD, maternal prepregnancy adiposity, and maternal third trimester IAUC. Additionally, prepregnancy adiposity and third trimester IAUC were regressed on maternal WSD, and third trimester IAUC was regressed on prepregnancy adiposity. Paths were also estimated from a variable capturing maternal age at birth to her prepregnancy adiposity, third trimester IAUC, and the measure of inflammation. The offspring behavioral measure was regressed on maternal WSD, prepregnancy adiposity, third trimester IAUC, and the measure of inflammation. Indirect effects were estimated using M*plus*’ *model indirect* command. Additionally, paths were estimated from offspring sex and offspring postweaning diet to the juvenile behavioral measure.

**Figure 2:**
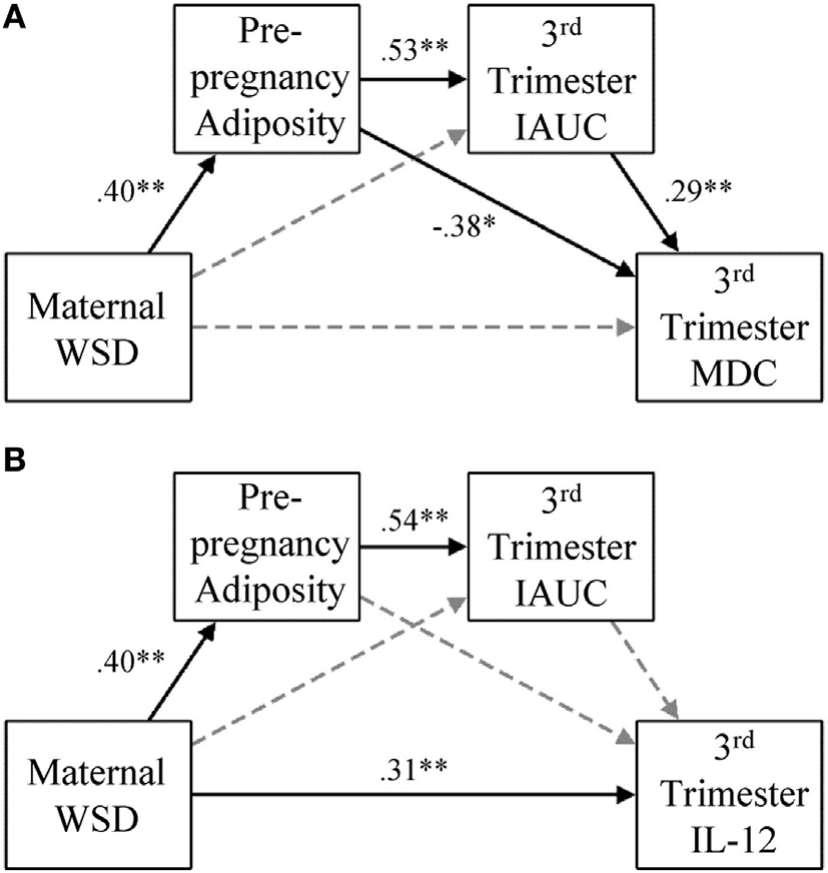
Gestational MDC and IL-12 response and degree of metabolic impairment. **(A)** In the presented model for maternal third trimester MDC, paths were also estimated from maternal age at birth to prepregnancy adiposity (β = 0.54, *p* < 0.001), third trimester IAUC (β = −0.02, *p* = 0.80), and maternal third trimester MDC (β = 0.03, *p* = 0.80), but were not visually depicted for ease of readability. **(B)** In the presented model for maternal third trimester, IL-12 paths were also estimated from maternal age at birth to prepregnancy adiposity (β = 0.54, *p* < 0.001), third trimester IAUC (β = −0.03, *p* = 0.74), and maternal third trimester IL-12 (β = −0.08, *p* = 0.58), but were not visually depicted for ease of readability. Black lines indicate significant direct effects (*p* < 0.05) and are labeled with β value. See results for further details on model fit and indirect effects. Gray dashed lines indicate paths that were estimated but were not statistically significant. WSD, Western-style diet; IAUC, insulin area under the curve; IL, interleukin. *p < 0.05 and **p < 0.01.

Main effects regression analysis revealed only one inflammatory variable was associated with offspring behavior and therefore was the only inflammatory marker subsequently tested in the aforementioned SEMs. *Post hoc* analysis was necessary to gain a broader understanding of how maternal diet and metabolic impairments effect peripheral inflammatory markers during pregnancy. Follow-up SEMs were used to investigate the associations among maternal WSD, metabolic variables, and inflammation (see Figure 2 for a visual depiction of these models). This was tested using individual SEMs for each investigated aspect of maternal third trimester inflammation. These tests used the same regression patterns described above but did not include offspring behavior categories.

## Results

### Inflammation Data Reduction

Prior to hypothesis testing, we conducted three confirmatory factor analyses (described above) to determine if it was statistically sound to combine the inflammatory marker variables to create an inflammatory burden latent variable, a cytokine latent variable, and a chemokine latent variable. Both the inflammatory burden latent variable [χ^2^ (62) = 84.30, *p* = 0.18, CFI = 0.96, TLI = 0.93, RMSEA = 0.06] and chemokine latent variable [χ^2^ (7) = 9.50, *p* = 0.22, CFI = 0.98, TLI = 0.94, RMSEA = 0.06] fit the data well, suggesting that these are appropriate ways to consider these variables. Factor loadings associated with both latent variables appear in Table 2. The cytokine latent variable did not fit the data adequately [χ^2^ (2) = 23.66, *p* = 0.00, CFI = 0.80, TLI = 0.11, RMSEA = 0.30] and thus was not included in further analyses. Because the inflammatory burden and chemokine latent variables were produced using non-nested models that consider different indicators, it was not possible to test whether one fits the data better than the other. Instead, we proceeded with analyses using both latent variables in order to examine different aspects of inflammatory response.

Results from the regression models used to relate maternal WSD to each of the individual protein markers are available in Table 2. Maternal third trimester MDC was associated with maternal WSD (β = −0.24, *p* = 0.012; mean ± SE: CTR = 481.99 ± 66.11 pg/mL, WSD = 287.06 ± 28.17 pg/mL). Gestational IL-12 was also associated with maternal WSD (β = 0.26, *p* = 0.002; mean ± SE: CTR = 252.46 ± 22.32 pg/mL, WSD = 512.74 ± 117.30 pg/mL). Of the bivariate correlations with offspring behavioral categories, only MDC correlated with any offspring behavior (*p* < 0.01, data not shown). Based on these preliminary analyses, third trimester MDC and IL-12 were selected as the two individual inflammatory markers to be considered in further SEM analysis.

### Modeling Perinatal Environment and Offspring Behavior

Results from the main effects regression models used to select the inflammation variable(s) to be used in behavioral models are presented in Table S1 in Supplementary Material. When considered in a model with maternal age at birth, maternal third trimester MDC was the only measure of inflammation that was associated with offspring behavior. Specifically, reduced maternal MDC was associated with increased engaged behaviors (β = −0.37, *p* = 0.001) and increased high-energy outbursts (β = −0.48, *p* = 0.001). As third trimester inflammatory burden, chemokine profile, and IL-12 were not directly associated with offspring behavior, MDC was the only inflammatory variable considered in subsequent SEMs.

Figure 1 presents the results from the SEMs used to test perinatal physiological influences on offspring behavior, controlling for maternal age at birth, offspring sex, and offspring postweaning diet. The model for offspring reactive anxiety fit the data well, χ^2^ (11) = 10.72, *p* = 0.47, CFI = 1.00, TLI = 1.01, RMSEA = 0.00. When maternal dietary, metabolic, and inflammatory variables were considered in a model together, maternal WSD was associated with greater reactive anxiety (β = 0.26, *p* < 0.01). Reactive anxiety was also inversely associated with offspring postweaning diet (β = −0.21, *p* = 0.02). Maternal WSD was additionally associated with greater ritualized anxiety (β = 0.23, *p* = 0.04); model fit for this SEM was adequate [χ^2^ (11) = 10.72, *p* = 0.46, CFI = 1.00, TLI = 1.01, RMSEA = 0.00]. The SEM for offspring high-energy outbursts also fit the data well, χ^2^ (11) = 12.67, *p* = 0.32, CFI = 0.99, TLI = 0.97, RMSEA = 0.03. Maternal prepregnancy adiposity (β = 0.30, *p* = 0.02) and third trimester MDC (β = −0.37, *p* = 0.009) exerted opposing effects on offspring high-energy outbursts. There was a significant indirect effect of maternal WSD on high-energy outbursts, *via* increased prepregnancy adiposity (β = 0.12, *p* = 0.03; 95% CI = 0.01, 0.24). The indirect effect of maternal WSD on high-energy outbursts *via* the prepregnancy adiposity→third trimester IAUC→MDC pathway was marginally significant (β = −0.03, *p* = 0.09; 95% CI = −0.05, 0.004). The model including offspring engaged behaviors provided good model fit, χ^2^ (11) = 12.02, *p* = 0.36, CFI = 0.99, TLI = 0.98, RMSEA = 0.02. Maternal third trimester MDC was inversely associated with offspring engaged behaviors (β = −0.34, *p* = 0.03) with no significant indirect effects. Although the model of offspring inactive behavior fit the data adequately [χ^2^ (11) = 10.67, *p* = 0.47, CFI = 1.00, TLI = 1.01, RMSEA = 0.00], there were no significant effects on offspring inactive behavior.

### Modeling Maternal Inflammation During Gestation

In each of the above models, the pattern of findings among maternal diet, age, metabolic variables, and MDC remained unchanged and are consistent with the *post hoc* model tested in the absence of behavior variables. Four SEMs were used to characterize the associations among maternal WSD, maternal prepregnancy adiposity, maternal third trimester IAUC, and maternal third trimester inflammation measures. These SEMs followed paths outlined above and controlled for maternal age at birth. The four models were identical except that each considered a different measure of maternal third trimester inflammation (MDC, IL-12, chemokines, and inflammatory burden).

Figure 2A presents the results from the model that included maternal third trimester MDC. This model fit the data well, χ^2^ (1) = 1.14, *p* = 0.29, CFI = 1.00, TLI = 0.99, RMSEA = 0.03. Maternal WSD was directly associated with greater prepregnancy adiposity (β = 0.40, *p* < 0.001) and indirectly associated with greater third trimester IAUC values *via* increased prepregnancy adiposity (β = 0.21, *p* < 0.001; 95% CI = 0.10, 0.33). WSD had an indirect effect on MDC *via* the prepregnancy adiposity to third trimester IAUC pathway (β = 0.06, *p* = 0.026; 95% CI = 0.01, 0.12), but the indirect effect of WSD on MDC *via* prepregnancy adiposity alone was only marginally significant (β = −0.15, *p* = 0.087; 95% CI = −0.33, 0.02).

The results from the model that considered IL-12 are presented in Figure 2B. This model fit the data well, χ^2^ (1) = 1.14, *p* = 0.29, CFI = 1.00, TLI = 0.98, RMSEA = 0.03. In addition to a direct effect on prepregnancy adiposity (β = 0.40, *p* < 0.001), WSD had a direct effect on IL-12 (β = 0.31, *p* = 0.001). Prepregnancy adiposity and third trimester IAUC were not associated with maternal third trimester IL-12 levels. The only significant indirect effect in this model was that from maternal WSD to third trimester IAUC, *via* prepregnancy adiposity (β = 0.21, *p* < 0.001; 95% CI = 0.10, 0.33).

**Figure 3:**
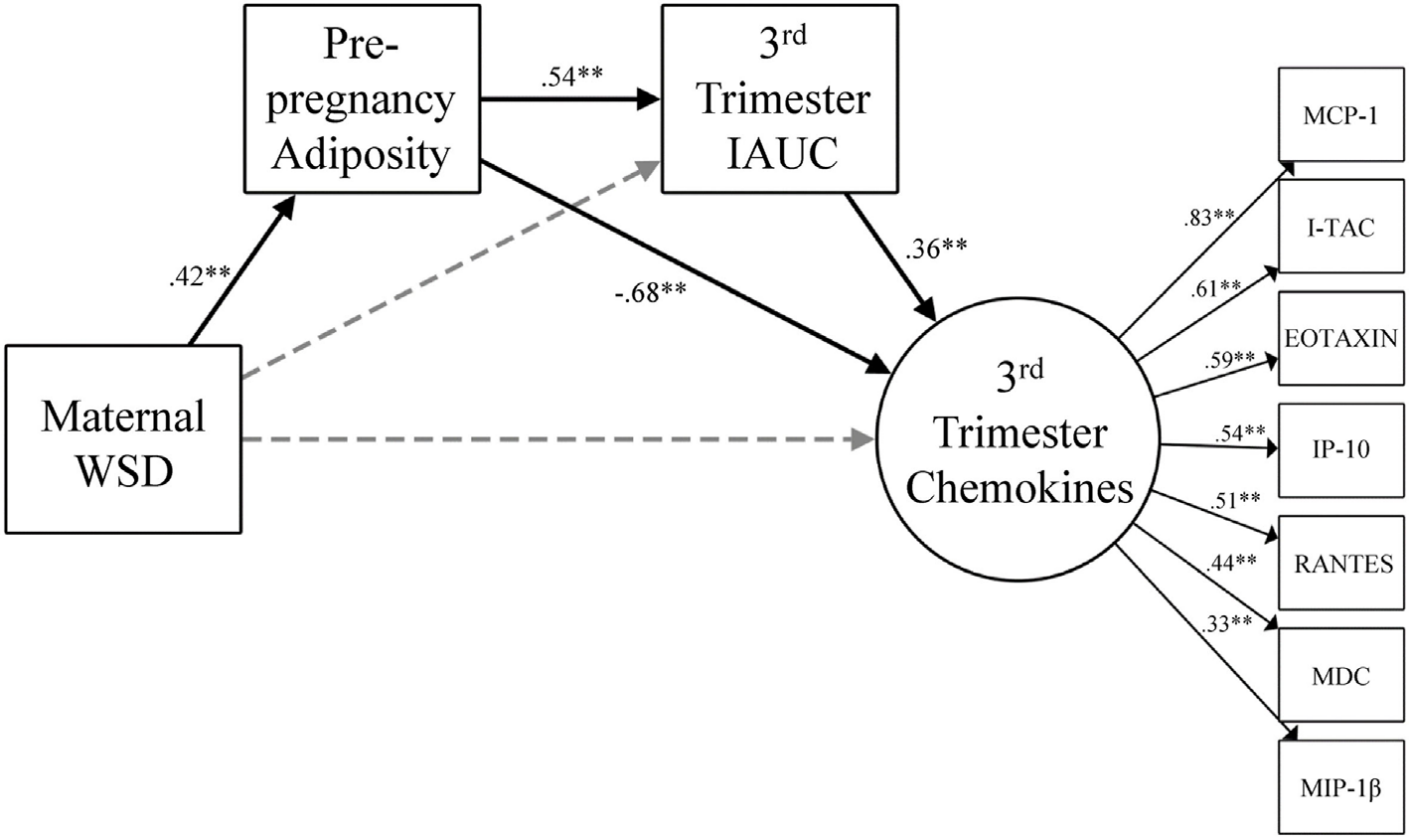
Gestational chemokine response and degree of metabolic impairment. The model for chemokine latent variable is presented with constituent chemokine protein markers and the corresponding standardized factor loadings. Paths were also estimated from maternal age at birth to prepregnancy adiposity (β = 0.53, *p* < 0.001), third trimester IAUC (β = −0.02, *p* = 0.83), and maternal third trimester chemokines (β = 0.24, *p* = 0.04) but were not visually depicted for ease of readability. Black lines indicate significant direct effects (*p* < 0.05) and are labeled with β value. See results for further details on model fit and indirect effects. Gray dashed lines indicate paths that were estimated but were not statistically significant. WSD, Western-style diet; IAUC, insulin area under the curve. *p < 0.05 and **p < 0.01.

Figure 3 presents the results from the model that considered maternal third trimester chemokines. This model also fit the data well [χ^2^ (32) = 44.85, *p* = 0.07, CFI = 0.94, TLI = 0.90, RMSEA = 0.05] and yielded the same pattern of findings as the one that included MDC. Maternal WSD was directly associated with greater prepregnancy adiposity (β = 0.42, *p* < 0.001) and, *via* increased prepregnancy adiposity, was indirectly associated with third trimester IAUC (β = 0.23, *p* < 0.001; 95% CI = 0.11, 0.34). Maternal WSD had opposing indirect effects on third trimester chemokines *via* prepregnancy adiposity (β = −0.29, *p* < 0.001; 95% CI = −0.43, −0.15), as well as *via* the prepregnancy adiposity to third trimester IAUC pathway (β = 0.08, *p* < 0.01; 95% CI = 0.03, 0.14).

The model that considered the maternal inflammatory burden latent variable did not fit the data adequately [χ^2^ (115) = 193.24, *p* = 0.00, CFI = 0.88, TLI = 0.84, RMSEA = 0.07], and thus the results are not detailed here (but are available by request from authors).

## Discussion

We hypothesized that WSD-induced changes in maternal metabolic state and gestational inflammation would increase stress-induced behavior in offspring during temperament assessment. Additionally, we posited that maternal WSD consumption alone would alter the maternal inflammatory profile to which offspring were exposed during prenatal development and independently impact offspring behavioral response. For the first time, we are able to report that maternal plasma levels of MDC (CCL22) were significantly associated with altered offspring behavior. Strikingly, maternal prepregnancy adiposity and WSD consumption each exhibited unique impacts on offspring behavior, without the involvement of maternal inflammatory response.

### WSD and Maternal Obesity Differentially Program Risk of Abnormal Offspring Behavior

Gestational and early postnatal exposure to WSD augmented typical and atypical stress behaviors in 11-month-old Japanese macaque offspring. WSD increased reactive anxiety, a behavioral composite representing species-typical displacement and anxious behaviors, mostly characterizing defensive behaviors in response to fear or stress (47). Excessive anxiety response can be maladaptive and suggests behavioral dysregulation in animals and humans (47, 48). Maternal WSD exposure further disrupted offspring behavioral response by increasing ritualized anxiety behaviors. These restrictive and repetitive behaviors are abnormal in all contexts and are indicative of anxiety response as well as ASD symptomology (49). This is the first study to isolate the behavioral programming effects of chronic WSD diet from maternal metabolic condition, demonstrating perinatal WSD alone induced long-term behavioral dysregulation in non-human primate offspring.

Despite the well-established link between WSD and obesity in our non-human primate model and the human population, maternal prepregnancy adiposity was independently associated with increased high-energy, explosive behaviors. The high-energy outburst variable was created due to the increased prevalence of several unique behaviors which do not fit typical descriptions of anxious behavior in non-human primates. Behaviors included in this variable commonly cooccurred and displayed similar qualities; they were intense, brief, borderline self-injurious, and typically occurred in the absence of triggering, stressful stimuli.

Elevated prepregnancy adiposity contributed to increased high-energy outbursts in 11-month-old offspring, mediating the effect of maternal WSD. Intense, self-aggressive outbursts are prolific in children and young adults with neuropsychiatric disorders (50–52). Impulse control disorders like intermittent explosive disorder are associated with obesity and diabetes in adults (53, 54), and our findings suggest the association may be cross-generational. Impulsive, explosive outbursts are typical of intermittent explosive disorder and bipolar disorder, both characterized by high levels of disruptive behaviors (55, 56).

Our results provide evidence that adiposity and WSD exhibit programming effects on unique areas of emotional and behavioral regulation. Perinatal WSD exposure predisposes offspring to exaggerated anxiety response and repetitive behaviors, while prepregnancy adiposity increases risk of impulsive and disruptive behaviors.

### Maternal MDC Influences Offspring Explosive Outbursts

Perhaps the most novel finding of the present study is the influence of maternal MDC on offspring behavior. Elevated levels of impulsive, high-energy outbursts, and engaged behaviors were associated with low maternal MDC at the third trimester. The engaged behavior category comprises species-typical responses to novelty and stimulation that are not considered indications of a negative affect. Some of the outburst behaviors, particularly escape and cage bite, appear to be extreme manifestations of common engaged behaviors, tactile and oral exploration, respectively. These two behavioral categories are highly correlated (*r* = 0.508, *p* < 0.001) and this overlap makes it difficult to statistically determine the extent to which there are unique or overlapping effects between the behaviors. Although high-energy outbursts and engaged behaviors are conceptually distinct, the results should be interpreted in light of this limitation.

#### MDC: Balance Between Pregnancy Viability and Fetal Inflammation?

Our examination of gestational influences on offspring behavioral development indicated that low maternal MDC contributes to altered behavioral response. Unlike other more commonly investigated inflammatory markers (e.g., IL-1β, TNF-α, and IL-6), to date no studies have been conducted examining the effect of maternal MDC on any offspring outcome in the fetal, early postnatal, or later developmental periods. We believe that the functions of MDC during pregnancy are key to the observed involvement in offspring behavioral regulation.

Macrophage-derived chemokine is an important indicator of anti-inflammatory response, perpetuating M2-polarized profiles, directing Treg cells, and preventing autoimmunity (57). These functions are of paramount importance during pregnancy, as half of the fetus’ antigens are foreign to the mother. MDC levels of pregnant women are threefold lower than non-pregnant women, and peripheral concentrations decline with gestational age (58, 59). MDC is implicated in aberrant pregnancy outcome, as decidua from spontaneous abortions and recurring miscarriages show elevated MDC and Treg infiltration (60). Although MDC is produced by both maternal and fetal-derived tissue, maternal decidual cells appear to be responsible for these autoimmune rejections.

Clearly, low maternal MDC is important for pregnancy viability, however, the resulting immunosuppression could have negative impacts on offspring inflammatory response. The fetal immune system can be activated by an immunocompromised maternal environment, and fetal-derived placental tissue can modulate MDC production following infection (61). In the central nervous system, offspring microglia are able to regulate MDC expression *in utero* in response to maternal toxin administration or oxidative stress (62, 63). Early postnatal studies in mice further demonstrate the importance of MDC to the developing neuroinflammatory response, as microglial expression patterns change in the first few days of development in healthy and immunocompromised animals (64, 65). Since fetal MDC expression can be modified by maternal factors during gestation, aberrant maternal MDC could indicate or induce altered offspring inflammatory response. We performed correlations between maternal third trimester plasma MDC and offspring plasma MDC at 13 months of age, and found that they were significantly associated (*r* = 0.267, *p* = 0.018). Future studies should investigate the impact of maternal MDC levels during gestation on fetal and juvenile peripheral and central inflammation.

#### MDC: Neuropathology Biomarker or Mechanism?

The present findings are the first to demonstrate that maternal MDC was associated with abnormal behavioral response in offspring. These results are supported by a number of clinical and animal studies which have found links between MDC levels and neuropsychiatric disorders. Peripheral MDC is positively associated with severity of ASD (66), schizophrenia (67–69), interpersonal sensitivity, and phobia symptomology (70). MDC is implicated in altered gaze perception bias and accuracy in patients with non-delusional schizophrenia (71). Self-directed gaze bias is an aspect of social cognition that is similarly impaired in individuals with bipolar disorder (72), a condition which likewise has been associated with aberrant MDC (73).

Despite the number of studies suggesting MDC is an important biomarker for neural health, relatively little is known about the impact of blood MDC levels on neural outcomes. Within neuropsychiatric disorders, neural correlates are limited to a study in first episode psychosis patients, where serum levels of MDC were significantly elevated and detrimental to white matter integrity (74). In contrast, plasma MDC in Alzheimer’s patients was irrelevant to disease symptomology; rather, elevated cerebrospinal fluid MDC levels predicted improved cognitive scores in treated individuals (75). Peripheral MDC is reduced in glioma cases, and in this neuroinflammatory state elevated MDC acted as survival-promoting factor (76). Whether high levels of peripheral MDC serves a detrimental or protective function seems to be dependent on the specifics of the neuropathology. Further research is required to investigate the association between peripheral MDC levels and central MDC availability, expression, and function. The function of MDC is best established in autoimmune neuropathologies, where MDC in cerebrospinal fluid and microglial MDC expression are associated with symptom severity and enhanced accumulation of peripheral mononuclear cells in the CNS (65, 77–79). Although considerably more research is needed to investigate the role of MDC in other developmental, psychiatric, and degenerative neuropathologies, MDC provides a promising target for potential screening or treatment options.

### Systematic Metabolic Impairment Moderates Gestational Inflammatory Response

We hypothesized that inflammation would mediate the effects of maternal diet and metabolic state on offspring behavior. Despite the fact that maternal diet, metabolic state, and MDC each contributed to aberrant behavioral regulation, this hypothesis was not supported. Although there were no significant indirect behavioral effects *via* MDC, the extent of metabolic impairment during gestation differentially altered maternal inflammatory response.

We confirmed that WSD and age contributed to elevated prepregnancy adiposity, which amplified insulin resistance during the third trimester. WSD indirectly increased insulin resistance through elevated adiposity but did not directly increase IAUC. This is consistent with the well-established body of literature indicating the significant endocrine function of adipose tissue and its contribution to dysregulated insulin response (80). We proposed that proinflammatory response would predominate in obese mothers with normal insulin tolerance (early metabolic impairment), but that anti-inflammatory response would prevail in mothers with obesity-induced insulin resistance (advanced metabolic impairment).

Mothers in both diet groups whose metabolic state was characterized by elevated adiposity with normal insulin tolerance exhibited suppressed circulating MDC and chemokine profile. Importantly, we found that plasma chemokines were conversely elevated in obese mothers with gestational insulin resistance. Obesity suppressed circulating third trimester chemokines and MDC, yet obesity-induced gestational insulin resistance elevated these measures, with WSD consumption contributing to both states. This supports our hypothesis that divergent inflammatory profiles resulted from progressing metabolic impairment, here characterized by the presence of insulin resistance, likely influenced by ATMs.

In addition to classical M1 and alternative M2 activations, ATMs can to be metabolically activated by free fatty acid levels in an interferon-independent pathway (29). Metabolically activated ATMs generate a single macrophage phenotype with dual-functions of proinflammatory cytokine production and anti-inflammatory lipid metabolism. The build-up of free fatty acids within metabolically activated ATMs is believed to facilitate this transition. Apoptotic adipocytes, prevalent in diet-induced obesity, increase levels of extracellular free fatty acids, enhancing the demand for ATM lipid metabolism, and advancing local insulin resistance (30). As pregnancy progresses, adipose tissue distribution changes to accommodate the growing fetus, and we posit that differences in redistribution between normal-weight and obese women (81) contribute to intensified adipocyte death and exacerbate local and systemic inflammatory response. Follow-up investigation should explore gestational weight change and alterations in adipokines like leptin in order to test how adipose tissue function changes across pregnancy.

Apart from the indirect effects WSD exhibited on maternal inflammatory response *via* diet-induced obesity and insulin resistance, WSD alone increased third trimester IL-12 levels. In accordance with established research, we demonstrate that chronic consumption and metabolism of a processed, high-fat, high-sugar diet increased proinflammatory response (25). IL-12 is the predominant cytokine in proliferating M1 pathways and is an important part of the proinflammatory feedback loop (82). Despite the significance of IL-12, a generalized proinflammatory profile was not observed as our cytokines did not group well in confirmatory factor analysis. This finding was intriguing; the component inflammatory factors (IFN-γ, TNF-α, MIF, IL-12, IL-1β, IL-1RA, and IL-6) are highly integrated and share common expression pathways (83). Furthermore, overall inflammatory burden did not fit our model of metabolic impairment during pregnancy. These findings could be due to the absence of inflammatory markers that were excluded based on our criteria of >80% samples above LLOQ. Additionally, pregnancy is an extreme state that does not represent the physiological norm, and these unique demands could contribute to altered inflammatory pathways in order to protect the fetus and mother (32). Thus, future studies should examine the prepregnancy inflammatory environment as an additional predictive factor, allowing for a specific focus on how pregnancy, maternal diet, and metabolic state alter prevailing inflammatory response.

### Conclusion

In our examination of maternal inflammation during pregnancy, MDC was of stand-alone importance for predicting offspring behavioral outcome. Of the component proteins, MDC was not the highest loading factor contributing to the chemokine latent variable, yet it was the only one that was significantly associated with alterations in offspring behavior. Thus, our findings suggest that the individual impact of a single inflammatory marker can be vital in programming offspring behavioral development. Our results support the current scientific opinion that strict classifications of pro- vs. anti-inflammatory, or M1 vs. M2 profiles can be overly simplistic and imprecise. Despite the highly homogenous profile of cytokines constituting our cytokine latent variable, this grouping was not statistically supported. Rather, IL-12 showed individual importance in diet-induced inflammation. Future investigation will attempt to examine the individual effect of all measured inflammatory markers. Of note, all 15 inflammatory factors analyzed in this study combined well into a single inflammatory burden variable, which was not associated with offspring behavior or maternal metabolic environment. We believe our current findings highlight the importance of dually investigating the overall inflammatory environment as well as individual inflammatory factors.

Inflammation, as addressed in this study, did not mediate the effects of maternal metabolic state on offspring behavior. However, we were able to provide evidence that diet-induced obesity and obesity-induced insulin resistance substantially and differentially altered the chemokine inflammatory response during gestation. Our results are in agreement with a metabolically activated inflammatory response, wherein prolonged exposure to elevated dietary fat and obesity state increases factors promoting adipocyte cell death, stimulating insulin resistance, and heightening the need for anti-inflammatory ATM function. The aim of this manuscript was to further identify developmental influences on offspring behavior, and so we focused on third trimester measures of inflammation. Having established the significance of prepregnancy condition on gestational inflammatory and metabolic state, it will be important to explore the effects of additional prepregnancy measures such as inflammation and glucose and insulin metabolism.

Other maternal factors, like infant care behaviors, can influence offspring neurodevelopment (84) and have been associated with maternal metabolic health (85) and more recently inflammation (86). The mediating effect of maternal behavioral measures ought to be considered in future studies. This study controlled for a number of maternal variables often accounted for in clinical models, however, there were some methodological constraints unique to animal models. In order to obtain the physiological samples required to measure inflammation and metabolic state, our model required gestational samples to be collected under sedation. Inducing anesthesia is often necessary when studying longitudinal animal models, and while every effort is taken to limit these events and control for the effects of anesthetic procedures, they remain a limitation of the model. It is also important to note that there is no single “metabolic state” variable. Although we investigated factors which are major indicators of metabolic health, prepregnancy adiposity and third trimester insulin response do not fully represent the complexity of metabolic disorders.

Behaviorally, we were able to further classify previously reported alterations in anxious behavior in 11-month-old Japanese macaques (40). We found that maternal WSD alone increased defensive and ritualized behaviors. Prepregnancy adiposity, whether diet-induced or otherwise, increased explosive outbursts. Neither of these influences were mediated by insulin response or inflammation. Independently, low maternal MDC increased impulsive and disruptive behaviors. Supporting the previously reported influence of postweaning WSD exposure on offspring behavior, this study confirmed that postweaning WSD suppressed adaptive stress response behaviors. It was hypothesized that exposure to WSD-induced chronic inflammation *in utero* could be a factor contributing to the differential anxiety presentation in maternal vs. postweaning WSD groups. However, the current findings do not support this hypothesis. Offspring behavior was only examined at 11 months of age in this study, and in future studies our group aims to evaluate behavioral response to temperament assessment in the later juvenile period. We also intend to investigate juvenile social behavior, as the restricted interests (reduced exploration and interaction with novel objects) and ritualized behaviors seen at 11 months suggest a potential ASD-like phenotype (40). In addition, we previously observed that yearling offspring exposed to a maternal WSD displayed altered TPH2 expression in the dorsal raphe and decreased dopamine fiber density in the prefrontal cortex, even after intervention of a control diet at weaning (40, 87). In order to provide further evidence of neuropsychiatric impairments, future studies will utilize the presented modeling techniques to investigate the gestational influences on serotonergic and dopaminergic development. Our present findings indicate that maternal diet, adiposity, and MDC each significantly influenced offspring emotional and behavioral dysregulation. Additional studies can build on these findings to improve prepregnancy interventions in hopes to find realistic ways to reduce the risk of offspring neuropsychiatric disorders.

## Ethics Statement

All animal procedures were in accordance with National Institutes of Health guidelines on the ethical use of animals and were approved by the Oregon National Primate Research Center (ONPRC) Institutional Animal Care and Use Committee.

## Author Contributions

ES conceived the project; JT, ES, and HG designed the research; JT, MD, DT, JB, TD, and ES performed the experiments; HG, JT, and ES analyzed the data; all authors discussed the data; JT, HG, ES, and MD wrote the manuscript, with contributions from all authors.

## Conflict of Interest Statement

The authors declare that the research was conducted in the absence of any commercial or financial relationships that could be construed as a potential conflict of interest.
