# Supplementary material for: Maternal Diet, Metabolic State, and Inflammatory Response Exert Unique and Long-Lasting Influences on Offspring Behavior in Non-Human Primates
Source: Front Endocrinol (Lausanne). 2018 Apr 23;9:161. doi: 10.3389/fendo.2018.00161 (PMC5924963; doi:10.3389/fendo.2018.00161)
Supplement: Supplementary file 1 [file table_1.docx]

Supplementary Material

**Maternal Diet, Metabolic State, and Inflammatory Response Exert Unique and Long-lasting Influences on Offspring Behavior in Non-human Primates**

Jacqueline R. Thompson^1,2^, Hanna C. Gustafsson^3^, Madison DeCapo^1,2^, Diana L. Takahashi^2^, Jennifer L. Bagley^1,2^, Tyler A. Dean^2^, Paul Kievit^2^, Damien A. Fair^3,4^, Elinor L. Sullivan^1,2,3,5*^

*** Correspondence:** Dr. Elinor L. Sullivan: sullivel@ohsu.edu

# Supplementary Figures and Tables

|  | Reactive Anxiety | | |  | Ritualized Anxiety | | |  | High-Energy Outbursts | | |  | Engaged Behaviors | | |  | Inactive Behaviors | | |
| --- | --- | --- | --- | --- | --- | --- | --- | --- | --- | --- | --- | --- | --- | --- | --- | --- | --- | --- | --- |
| Maternal Measures | β | SE | *p value* |  | β | SE | *p value* |  | β | SE | *p value* |  | β | SE | *p value* |  | β | SE | *p value* |
| Third Trimester Inflammatory Burden | -.19 | .14 | .169 |  | -.04 | .11 | .707 |  | -.09 | .14 | .488 |  | .01 | .14 | .965 |  | .19 | .16 | .246 |
| Dam Age at Birth | -.23 | .15 | .127 |  | -.19 | .14 | .168 |  | .01 | .10 | .948 |  | -.14 | .13 | .296 |  | .09 | .11 | .409 |
| Third Trimester Chemokines | -.22 | .14 | .101 |  | .06 | .11 | .574 |  | -.14 | .17 | .425 |  | -.06 | .16 | .693 |  | .11 | .12 | .366 |
| Dam Age at Birth | -.23 | .15 | .134 |  | -.17 | .13 | .202 |  | .00 | .10 | .964 |  | -.14 | .13 | .264 |  | .07 | .11 | .491 |
| Third Trimester MDC | -.20 | .17 | .229 |  | -.09 | .09 | .294 |  | -.48 | .13 | .000** |  | -.37 | .11 | .001** |  | .13 | .07 | .082 |
| Dam Age at Birth | -.21 | .15 | .151 |  | -.19 | .13 | .144 |  | -.02 | .10 | .863 |  | -.17 | .13 | .184 |  | .07 | .10 | .489 |
| Third Trimester IL-12 | .00 | .08 | .957 |  | -.05 | .06 | .400 |  | .04 | .09 | .634 |  | .11 | .12 | .338 |  | .03 | .16 | .852 |
| Dam Age at Birth | -.20 | .16 | .224 |  | -.19 | .14 | .152 |  | .03 | .11 | .756 |  | -.10 | .14 | .456 |  | .07 | .12 | .573 |

## Supplementary Table 1. Main effects models.

Presented are the results from the main effects models used to test the association between the maternal inflammatory variables and the offspring behaviors. The results of the models were used to inform which metrics of inflammation were included in structural equation models used to test the influences on offspring behavior. SE= standard error of the estimate. **p* < .05, ***p* < .01.
